# Coexistence of WiFi and WiMAX Systems Based on PS-Request Protocols^†^

**DOI:** 10.3390/s111009700

**Published:** 2011-10-13

**Authors:** Jongwoo Kim, Suwon Park, Seung Hyong Rhee, Yong-Hoon Choi, Young-uk Chung, Ho Young Hwang

**Affiliations:** 1 Department of Electronics and Communications Engineering, Kwangwoon University, Seoul 139-701, Korea; E-Mail: jongwoo_kim@kw.ac.kr; 2 Department of Electronics Convergence Engineering, Kwangwoon University, Seoul 139-701, Korea; E-Mail: rhee@kw.ac.kr; 3 Department of Information Control Engineering, Kwangwoon University, Seoul 139-701, Korea; E-Mail: yhchoi@kw.ac.kr; 4 Department of Electronic Engineering, Kwangwoon University, Seoul 139-701, Korea; E-Mail: yuchung@kw.ac.kr; 5 Department of Computer Engineering, Kwangwoon University, Seoul 139-701, Korea; E-Mail: hyhwang@kw.ac.kr

**Keywords:** coexistence, mutual interference, power saving mode, PS-Request, duration field

## Abstract

We introduce both the coexistence zone within the WiMAX frame structure and a PS-Request protocol for the coexistence of WiFi and WiMAX systems sharing a frequency band. Because we know that the PS-Request protocol has drawbacks, we propose a revised PS-Request protocol to improve the performance. Two PS-Request protocols are based on the time division operation (TDO) of WiFi system and WiMAX system to avoid the mutual interference, and use the vestigial power management (PwrMgt) bit within the Frame Control field of the frames transmitted by a WiFi AP. The performance of the revised PS-Request protocol is evaluated by computer simulation, and compared to those of the cases without a coexistence protocol and to the original PS-Request protocol.

## 1. Introduction

Many wireless communication systems have been developed and deployed to support various kinds of wireless data services. Many of the developed frequency bands are becoming more congested, especially in the densely populated urban areas due to the scarcity of commercially usable frequency resources and the explosive increasing desire for wireless data service. Consequently, it is essential that several heterogeneous wireless communication systems share a frequency band if possible, such as the 2.4 GHz ISM (Industrial, Scientific, and Medical) band. The spectrum sharing of several heterogeneous systems is termed coexistence. Coexistence can improve the utilization of the frequency band, because all the participating systems are not fully using their frequency band. However, the coexistence of heterogeneous wireless communication systems sharing a spectrum simultaneously and in the same geographic region causes their mutual interference, the coexistence problem. This degrades the performance of each wireless communication system. Sharing in the unlicensed bands, including 2.4 GHz ISM band, has been studied for a long time. Well known wireless communication systems in the ISM band are WiFi for wireless local area network (WLAN) systems, Bluetooth or ZigBee for wireless personal area networks (WPANs), and the like.

**Figure 1.**
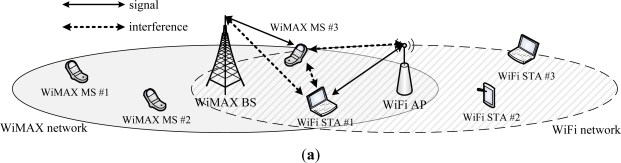

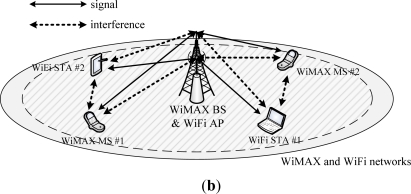
Coexistence of WiFi and WiMAX systems. (**a**) WiFi AP and WiMAX BS are not collocated; (**b**) WiFi AP and WiMAX BS are collocated.

There has been growing demand for broadband connections, along with the proliferation of wireless data services. WiMAX (Worldwide Interoperability for Microwave Access) for wireless metropolitan area network (WMAN) systems is an attractive technology to address this need. WiMAX is being considered as a candidate system operated in the ISM band, because it can provide low price or free high speed wireless internet service. If the WiMAX system is newly deployed in the ISM band, like a kind of home base station, it inevitably generates interference with the other wireless communication systems that exist in the ISM band. WiFi is a popular wireless communication system operated in that band, and deployed in many indoor or outdoor places. Therefore, the coexistence of WiFi and WiMAX systems sharing a frequency band should be studied, when they operate in an adjacent or the same area, as shown in Figure 1.

Table 1 defines the four types of coexistence of heterogeneous wireless communication systems. In this paper, we focus on the case in which WiFi system based on IEEE 802.11g and WiMAX system based on IEEE 802.16e are collaborative. We want to make them coexist, based on their minimum collaboration and the minimum change of their specifications.

In Section 2, we explain related work on coexistence. In Section 3, we introduce the time division operation of WiFi and WiMAX systems to avoid their mutual interference. In Sections 4 and 5, we introduce the previously proposed PS-Request protocol of the WiFi system and coexistence zone within the WiMAX frame structure. In Section 6, we propose a revised PS-Request protocol to resolve drawbacks of the original PS-Request protocol. In Section 7, we evaluate by simulation the performance of the revised PS-Request protocol. Finally, we will conclude in Section 8.

## 2. Related Work

Many studies have been conducted on the coexistence of heterogeneous wireless communication systems. The coexistence of WiFi and Bluetooth systems was dealt in [1–3], that of WiFi and ZigBee systems was discussed in [4,5]. Chiasserini [1] proposed two solutions for the coexistence of WiFi and Bluetooth systems. One involves WiFi STAs estimating the interference pattern, and transmitting a frame of the minimum payload size, 500 bytes, comparable to the duration of a single slot Bluetooth packet. This may reduce the probability of collisions between the two systems. However, it is hard to estimate the interference pattern. The second deals with frequency hopping, a suggested packet length and restricts the length of packets. Thus, the allowable data rate decreased. Golmie [2] introduced a backoff strategy and adaptive frequency hopping (AFH). This also reduced the collision probability of the two systems. The backoff strategy does not require changes to the Bluetooth specification. However, changing the frequency hopping pattern requires some changes to the Bluetooth specification. The benefits of AFH may not be as obvious for delay jitter and packet loss constrained applications, such as voice or video services. Hsu [3] studied a dynamic WiFi system fragmentation technique. A WiFi station (STA) divides one frame into several fragments, and sends them. The WiFi STA should be acknowledged fragment by fragment. If the transmission of a specific fragment fails, the WiFi STA only retransmits the failed fragment. This decreases the allowable data rate, because it reduces the length of the collided fragment. Sikora [4] evaluated the effects of the mutual interference, and quantity coexistence issues. Wei [5] introduced several coexistence models between ZigBee and WiFi systems, and evaluated their interference effects.

References [6–11] deal with the coexistence of WiFi and WiMAX systems. Dongeun Kim [6] proposed a solution based on a circulator to reduce the mutual interference between them. It showed a width of the overlapped frequency band to tolerate the mutual interference. Even though both systems can endure a certain amount of mutual interference, it does not avoid but rather mitigates the mutual interference. Berlemann [7,8] and Siddique [9] proposed solutions based on time division multiplexing (TDM) of WiMAX systems based on IEEE802.16 and WiFi systems based on IEEE802.11a/e. They improved spectral efficiency. However, no protocol controls the transmission of WiFi STAs. Even if a certain period is allocated to the WiFi system, a WiFi STA may send frames beyond the period. This can cause the mutual interference. Jing [10] studied the coexistence between WiMAX systems based on IEEE802.16a and WiFi systems based on IEEE802.11b. A WiFi access pointer (AP) senses WiFi channels and selects a channel with the minimum received signal strength indicator (RSSI). The WiFi AP broadcasts the selected WiFi channel, and all of WiFi STAs move to it. However, if an interferer is not located within the range of a WiFi AP, the WiFi AP cannot recognize it. Jing [11] also introduced a cognitive radio (CR) method. Each node announces information, such as frequency, power, modulation, duration, interference margin, and service type, through the common spectrum coordination channel. The method can reduce the influence of the mutual interference, but it needs much overhead. Two mechanisms termed Listen-Before-Talk (LBT) and Extended Quiet Period (EQP) are adopted to perform white space detection efficiently based on IEEE 802.16h [12]. A WiMAX BS should listen to a channel all the time to know whether the channel is occupied, and adopt LBT to determine if an EQP is needed after the end of a quiet period. If the channel is unoccupied, the WiMAX BS and WiMAX MSs transmit frames during an active period. The WiMAX BS and WiMAX MSs do not transmit during a quiet period. This information about the quiet period is included in the DL-MAP. It is extended if the channel is still occupied after the quiet period. Thus, it is termed Extended Quiet Period (EQP) in which there is no transmission. John [13] evaluated the performance of LBT and EQP protocols.

## 3. Time Division Operation of WiFi and WiMAX Systems

**Figure 2.**
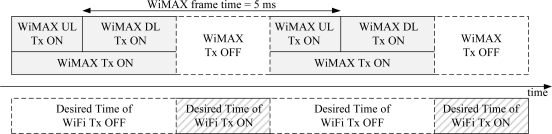
Desired TDO of WiFi and WiMAX systems.

We exploit the time division operation (TDO) to avoid or reduce the mutual interference between WiFi and WiMAX systems, as shown in Figure 2. The mutual interference can be avoided, since the TDO means that only one of two systems transmits, and the other system pauses its transmission [14,15]. Master devices of the systems, such as a WiFi AP and a WiMAX base station (BS), should control the signal transmission of its terminals, such as WiFi STAs and WiMAX mobile stations (MSs), respectively, to achieve the TDO, as shown in Figure 2. A little information exchange between WiFi and WiMAX systems is needed for timing synchronization of the transmission periods of two systems.

In this paper, we only focus on the coexistence of collaborative WiFi and WiMAX systems. Thus, we assume the minimum collaboration between a WiMAX BS and a WiFi AP for timing synchronization of the transmission period of each system is acquired. Information exchange among the WiMAX BS and WiFi STAs, among WiMAX MSs and the WiFi AP, and among WiMAX MSs and WiFi STAs, is unnecessary. However, the WiMAX BS and the WiFi AP should independently provide information about the transmission period to their terminals. If the WiMAX BS and the WiFi AP are in one device, as shown in Figure 1(b), the collaboration can be easily implemented.

A WiMAX BS based on IEEE802.16e specification can control the signal transmission of all of its WiMAX MSs through the DL-MAP (Downlink map) and UL-MAP (Uplink map). However, a WiFi AP based on IEEE802.11g cannot control the signal transmission of all its WiFi STAs. Thus, the desired structure of the TDO, as shown in Figure 2, cannot be achieved at the WiFi STAs, even though the WiFi AP can be operated based on the TDO structure due to the minimum information exchanged with the WiMAX BS. We propose a PS-Request protocol, the so called original PS-Request protocol, which makes the WiFi AP control the signal transmission of all of its WiFi STAs, to solve the problem.

## 4. Original PS-Request Protocol

**Figure 3.**
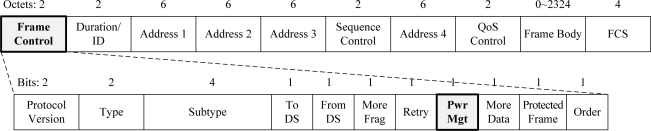
Format of general MAC frame of WiFi system.

Figure 3 shows the format of a general medium access control (MAC) frame of the WiFi system based on the IEEE802.11g specification. It comprises a set of fields that occur in a fixed order in all frames, as shown in Figure 3. The conventional WiFi system has three frame types: control, data, and management. Each of the frame types has several defined subtypes. The control frame consists of sixteen subtypes. Eight subtypes are being used, and eight subtypes are reserved. Detailed explanations of the types and subtypes are given in the IEEE802.11g specification [16].

The original PS-Request protocol is based on the use of the vestigial power management (PwrMgt) bit within the frames transmitted by a WiFi AP. In the case of an infrastructure configuration, the conventional WiFi system based on IEEE 802.11g specification can only support the power management mode for WiFi STAs. A WiFi STA can be in one of the two power management modes: an active mode (AM) or a power-saving mode (PSM). A WiFi STA in the AM is fully activated. It can send or receive frames at any time. A WiFi STA in the PSM can be in one of two states: a sleep state or an awake state. The WiFi STA in the PSM is usually in the sleep state, and periodically moves to the awake state to receive frames from a WiFi AP, such as beacon frames. The WiFi STA consumes much smaller energy than it does in the AM, because the WiFi STA in the PSM does not send any frames.

In the legacy WiFi system of an infrastructure configuration, a WiFi STA that wishes to move into the PSM shall inform its WiFi AP of an indication by setting the PwrMgt bit within the Frame Control field of the frame including the indication to ‘1’. The WiFi STA should be acknowledged by its WiFi AP, and move into the PSM. However, a WiFi AP cannot be in the PSM, because none of its WiFi STA can communicate for the PSM duration of the WiFi AP. Therefore, the PwrMgt bit within the Frame Control field of all frames transmitted by the WiFi AP is always set to ‘0’. This indicates that the WiFi AP is always in the AM.

If all WiFi STAs within the coverage of a WiFi AP are in the PSM, they do not transmit any signals. Thus, they do not generate interference to other systems during the PSM period. That is, if a WiFi system, including a WiFi AP and all of its WiFi STAs, do not transmit a signal during a specified period, the other wireless communication systems, such as a WiMAX system, can communicate without interference during the period. However, this can be achieved, only if the WiFi AP and all of its WiFi STAs move into the PSM. As mentioned before, the PSM of the conventional WiFi system can be controlled only by the WiFi STAs. That is, the WiFi AP cannot control the signal transmission of its WiFi STAs.

**Figure 4.**
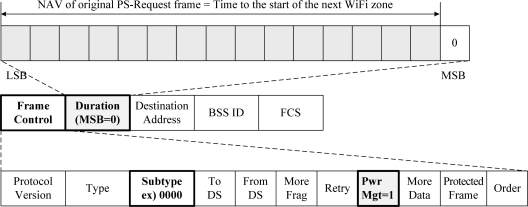
Format of original PS-Request frame.

**Figure 5.**
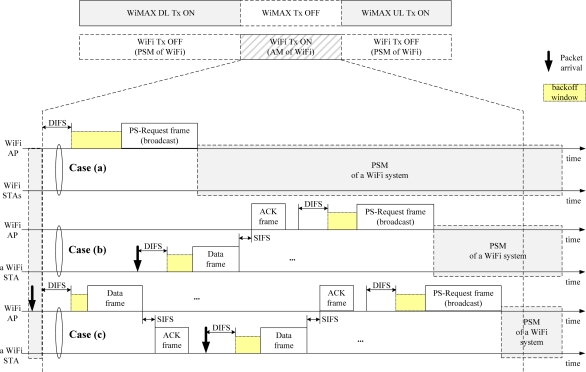
Example of the original PS-Request protocol.

We previously proposed to use the vestigial PwrMgt bit within the Frame Control field of frames transmitted by a WiFi AP for the WiFi AP to control the signal transmission of all of its WiFi STAs. We named it the PS-Request protocol. The PS-Request frame transmitted by the WiFi AP commands all its WiFi STAs to move into the PSM to stop them transmitting a signal. Figure 4 shows a format of the proposed PS-Request frame. One of the eight reserved subtypes, such as 0000, can be allocated, and the PwrMgt bit is set to ‘1’ in the Frame Control field. The Duration/ID field represents the value of a network allocation vector (NAV). This means that the WiFi AP will not transmit a signal during the PSM period of the NAV, and the WiFi AP stops its WiFi STAs from transmitting a signal during the period. Figure 5 shows an example of the original PS-Request protocol. There are three cases after the start time of WiFi Tx ON period equal to the end time of WiMAX Tx ON period, as shown in Figure 5.

### 

#### Case (a):

A WiFi AP collaborating with a WiMAX BS senses a wireless medium during a distributed inter-frame space (DIFS) time. If the medium is idle, the WiFi AP begins the backoff procedure. When its backoff timer expires, the WiFi AP sends the proposed PS-Request frame. From then on, the PSM of all of WiFi STAs starts.

#### Case (b):

Before the transmission of the original PS-Request frame, a WiFi STA receiving a packet sends its data frame after the CSMA (Carrier Sense Multiple Access)/CA (Collision Avoidance) procedure of the WiFi system, and receives an ACK frame from its WiFi AP. Then, the WiFi AP sends the proposed PS-Request frame, as in Case (a). Then, the PSM of all WiFi STAs starts.

#### Case (c):

Before the transmission of the original PS-Request frame, a WiFi AP receiving a packet sends its data frame after the CSMA/CA procedure of the WiFi system, and receives an ACK frame from a corresponding WiFi STA. Then, a WiFi STA receiving a packet sends its data frame after the CSMA/CA procedure of the WiFi system, and receives an ACK frame from its WiFi AP, as in Case (b). Then, the WiFi AP sends the proposed PS-Request frame, as in Case (a). Then, the PSM of all WiFi STAs starts. All WiFi STAs are in the PSM without sending an ACK frame to its WiFi AP, because the PS-Request frame is broadcast. The value of the Destination Address field within the frames transmitted by the WiFi AP determines if it is unicast or broadcast.

## 5. Proposed Coexistence Zone within WiMAX Frame Structure

**Figure 6.**
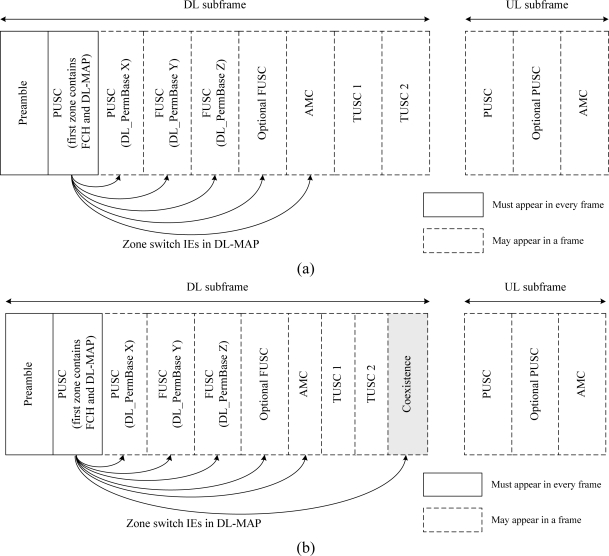
WiMAX zones. (**a**) Conventional WiMAX zones; (**b**) WiMAX zones, including the proposed coexistence zone.

There are already several zones in the WiMAX system, as shown in Figure 6(a). The IEEE802.16e specification provides detailed explanations of these zones [17]. They may exist in the WiMAX frame structure to support some particular functions for a WiMAX system. The DL PUSC (Partial Usage of Sub-Channel) zone for FCH and DL-MAP is mandatory, and the UL PUSC is the default. We proposed a new zone, termed the coexistence zone, as shown in Figure 6(b).

**Figure 7.**
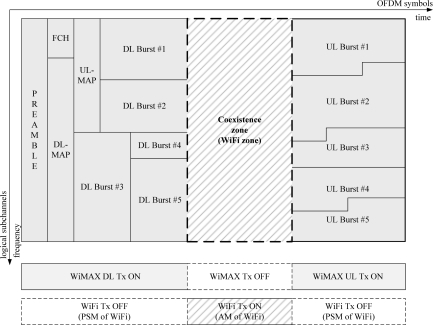
Proposed coexistence zone within WiMAX frame structure.

Figure 7 shows the coexistence zone within the WiMAX frame structure. During the coexistence zone, the WiMAX system does not transmit any signal, because no OFDM symbols and subcarriers should be used for communication. We temporarily call the coexistence zone the WiFi zone, for the coexistence of WiFi and WiMAX systems, because the coexistence zone is used for WiFi systems in this paper. The PSM and the AM of WiFi STAs are alternated, based on the original PS-Request frame transmitted by a WiFi AP, as shown in Figure 7. The WiMAX system pauses its signal transmission during the WiFi zone. Thus, either the WiFi AP or its WiFi STAs can send frames without interference from the WiMAX system according to the CSMA/CA procedure of the WiFi system. The WiFi AP sends the original PS-Request frame to all its WiFi STAs to protect the WiMAX UL and DL transmission ON time from signals from WiFi STAs.

## 6. Revised PS-Request Protocol

We proposed the PS-Request protocol, the so called original PS-Request protocol, to avoid the mutual interference between WiFi and WiMAX systems. However, we found that the original PS-Request protocol cannot completely solve the coexistence problem of WiFi and WiMAX systems. In the original PS-Request protocol, a WiFi AP only informs its WiFi STAs of the time to the start of the next WiFi zone, which is given in the Duration/ID field of the PS-Request frame, as shown in Figure 4. From this time, the WiFi AP and its WiFi STAs may compete to acquire the WiFi channel based on the CSMA/CA procedure of the WiFi system.

**Figure 8.**
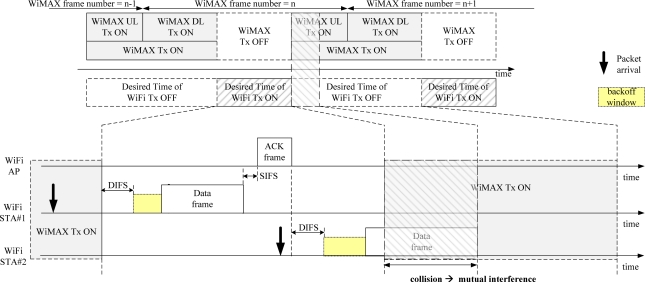
Drawback of the original PS-Request protocol.

As shown in Figure 5, the WiFi STAs receiving the original PS-Request frame from its WiFi AP cannot know the time remaining in the current WiFi zone. They only know the time to the start of the next WiFi zone. Thus, the remaining time of the current WiFi zone can be wasted. A WiFi STA that acquires a wireless medium after competition within the next WiFi zone, may send a frame beyond the next WiFi zone, as shown in Figure 8, because it does not know the time to the end of the next WiFi zone. In that case, the mutual interference may be inevitable. This degrades the performance of both WiMAX and WiFi systems.

**Figure 9.**
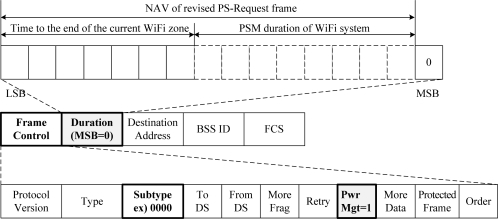
Format of revised PS-Request frame.

If WiFi STAs can know the remaining time of the current WiFi zone after receiving a PS-Request frame, they can decide to send frames either at a time or at several times by segmentation. By doing so, the mutual interference, as shown in Figure 8, can be avoided. Thus, we propose a revised PS-Request protocol that delivers both the time to the end of the current WiFi zone and the PSM duration to the WiFi STAs, as shown in Figure 9. The Duration/ID field in the revised PS-Request frame represents two values of NAV. One is the time to the end of the current WiFi zone, and the other is the PSM duration. The sum is equal to the time to the start of the next WiFi zone of the original PS-Request frame. In this paper, we assume that seven bits of fifteen bits represent the time to the end of the current WiFi zone and the other eight bits represent the PSM duration. The number of bits for each part is determined according to the desired resolution and maximum value of the time.

**Figure 10.**
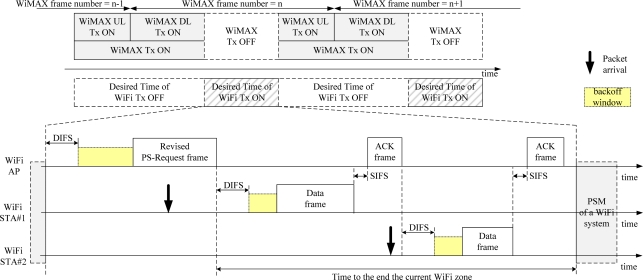
Example of the revised PS-Request protocol.

Figure 10 shows an example of the revised PS-Request protocol. A WiFi AP broadcasts a revised PS-Request frame through the transmission procedure of the WiFi system. WiFi STAs receiving the frame can send their frames until the time to the end of the current WiFi zone after a competition. After that time, all WiFi STAs are in the PSM, until the start of the next WiFi zone.

## 7. Simulation Results

### 7.1. System Assumption

As mentioned before, the proposed two PS-Request protocols operate based on an information exchange only between a WiFi AP and a WiMAX BS. They need the minimum collaboration between the WiFi AP and the WiMAX BS, such as the timing synchronization, to know the start and end times of the coexistence zone within the WiMAX frame structure. However, they do not need an information exchange among WiFi AP and WiMAX MSs, among WiFi STAs and WiMAX BS, and among WiFi STAs and WiMAX MSs.

### 7.2. Performance Measures

Throughput of each system is considered as one of the performance measures to be evaluated by the simulation [18].

$N_{\mathrm{WiFi}}^{\mathrm{bit}}$ and $N_{\mathrm{WiMAX}}^{\mathrm{bit}}$ are the total number of successfully transmitted data bits of WiFi and WiMAX systems, respectively. $N_{\mathrm{WiMAX},\mathrm{DL}}^{\mathrm{bit}}$ and $N_{\mathrm{WiMAX},\mathrm{UL}}^{\mathrm{bit}}$ are the total number of successfully transmitted WiMAX data bits through DL (Downlink) and UL (Uplink), respectively:

(1) $$N_{\mathrm{WiFi}}^{\mathrm{bit}}=\sum_{i=1}^{N_{\mathrm{STA}}}N_{\mathrm{WiFi}}^{\mathrm{ofdm}}\times 48\times M_{\mathrm{WiFi}}^{i}\times C_{\mathrm{WiFi}}^{i}$$

where *i* is the index of a WiFi STA and *N_STA_* is the number of WiFi STAs. $N_{\mathrm{WiFi}}^{\mathrm{ofdm}}$ is the number of transmitted OFDM symbols. Forty eight is the number of data subcarriers of 64 subcarriers for the WiFi system based on IEEE802.11g. $M_{\mathrm{WiFi}}^{i}$ and $C_{\mathrm{WiFi}}^{i}$ denote the modulation order and the code rate of WiFi STA of index *i*, respectively:

(2) $$N_{\mathrm{WiMAX},\mathrm{DL}}^{\mathrm{bit}}=\sum_{j=1}^{N_{\mathrm{MS}}}N_{\mathrm{WiMAX}}^{\mathrm{slot},j}\times 48\times M_{\mathrm{WiMAX}}^{j}\times C_{\mathrm{WiMAX}}^{j}$$

(3) $$N_{\mathrm{WiMAX},\mathrm{UL}}^{\mathrm{bit}}=\sum_{j=1}^{N_{\mathrm{MS}}}N_{\mathrm{WiMAX}}^{\mathrm{tile},j}\times 12\times M_{\mathrm{WiMAX}}^{j}\times C_{\mathrm{WiMAX}}^{j}$$

where *j* is the index of WiMAX MS, and *N_MS_* is the number of WiMAX MS’s. $M_{\mathrm{WiMAX}}^{j}$ and $C_{\mathrm{WiMAX}}^{j}$ denote the modulation order and the code rate for each data burst of WiMAX MS of index *j*, respectively. $N_{\mathrm{WiMAX}}^{\mathrm{slot},j}$ and $N_{\mathrm{WiMAX}}^{\mathrm{tile},j}$ denote the number of allocable resource units to the MS of index *j* for DL and UL, respectively. Forth eight and 12 denote the number of subcarriers in one slot and in one tile, respectively.

$N_{\mathrm{WiMAX}}^{\mathrm{frame}}$ is the number of WiMAX frames transmitted in a simulation. $T_{\mathrm{WiMAX}}^{\mathrm{frame}}$ is the frame time of the WiMAX system, and is typically *5* ms:

(4) $$T_{\mathrm{WiMAX}}^{\mathrm{frame}}=\left(N_{\mathrm{DL}}^{\mathrm{ofdm}}+N_{\mathrm{CZ}}^{\mathrm{ofdm}}+N_{\mathrm{UL}}^{\mathrm{ofdm}}\right)\times T_{\mathrm{symbol}}^{\mathrm{ofdm}}+T_{\mathrm{TTG}}+T_{\mathrm{RTG}}$$

$N_{\mathrm{DL}}^{\mathrm{ofdm}}$, $N_{\mathrm{CZ}}^{\mathrm{ofdm}}$ and $N_{\mathrm{UL}}^{\mathrm{ofdm}}$ denote the number of WiMAX OFDM symbols allocated to the DL subframe, the coexistence zone and the UL subframe within a WiMAX frame structure, respectively. $N_{\mathrm{DL}}^{\mathrm{ofdm}}$ includes one DL OFDM symbol for preamble, two DL OFDM symbols for FCH, DL-MAP and UL-MAP, and the other DL OFDM symbols for DL-Bursts of the WiMAX system. DL-Bursts of the WiMAX system are composed of DL OFDM symbols in multiples of two, and UL-Bursts of the WiMAX system are composed of UL OFDM symbols in multiples of three. $T_{\mathrm{symbol}}^{\mathrm{ofdm}}$ is one OFDM symbol duration of the WiMAX system. *T_TTG_* and *T_RTG_* denote the transmit/receive transition gap (TTG) and the receive/transmit transition gap (RTG) within a WiMAX frame structure, respectively.

For the performance evaluation of each system, the average throughputs of WiFi and WiMAX systems are defined, as follows:

(5) $$R_{\mathrm{WiFi}}≜\frac{N_{\mathrm{WiFi}}^{\mathrm{bit}}}{T_{\mathrm{WiFi}}^{\mathrm{TxON}}\times N_{\mathrm{WiMAX}}^{\mathrm{frame}}}=\frac{N_{\mathrm{WiFi}}^{\mathrm{bit}}}{N_{\mathrm{CZ}}^{\mathrm{ofdm}}\times T_{\mathrm{symbol}}^{\mathrm{ofdm}}\times N_{\mathrm{WiMAX}}^{\mathrm{frame}}}$$

(6) $$R_{\mathrm{WiMAX}}≜\frac{N_{\mathrm{WiMAX}}^{\mathrm{bit}}}{T_{\mathrm{WiMAX}}^{\mathrm{TxON}}\times N_{\mathrm{WiMAX}}^{\mathrm{frame}}}=\frac{N_{\mathrm{WiMAX},\mathrm{DL}}^{\mathrm{bit}}+N_{\mathrm{WiMAX},\mathrm{UL}}^{\mathrm{bit}}}{(N_{\mathrm{DL}}^{\mathrm{ofdm}}+N_{\mathrm{UL}}^{\mathrm{ofdm}})\times T_{\mathrm{symbol}}^{\mathrm{ofdm}}\times N_{\mathrm{WiMAX}}^{\mathrm{frame}}}$$

### 7.3. Simulation Environment

In this paper, WiMAX and WiFi systems are based on IEEE802.16e and IEEE802.11g specifications, respectively. For the simplicity of simulation and the performance evaluation of an extreme case, we assume that the WiMAX system consists of one WiMAX BS and only one WiMAX MS, and all of the radio resources are allocated to the WiMAX MS. The WiFi system also comprises one WiFi AP and one WiFi STA. Table 2 specifies the simulation parameters of the WiMAX system. Table 3 gives simulation conditions. The transmission period of each system is allocated based on the number of OFDM symbols of the WiMAX system.

For the case of $N_{\mathrm{DL}}^{\mathrm{ofdm}}+N_{\mathrm{CZ}}^{\mathrm{ofdm}}+N_{\mathrm{UL}}^{\mathrm{ofdm}}=42$ and $T_{\mathrm{symbol}}^{\mathrm{ofdm}}=115.2\mu s$, $T_{\mathrm{WiFi}}^{\mathrm{TxON}}=1.8432\;\text{ms}=16\times 115.2\mu s$ and $T_{\mathrm{WiMAX}}^{\mathrm{TxON}}=2.9952\;\text{ms}=26\times 115.2\mu s$.

### 7.4. Simulation Results

**Figure 11.**
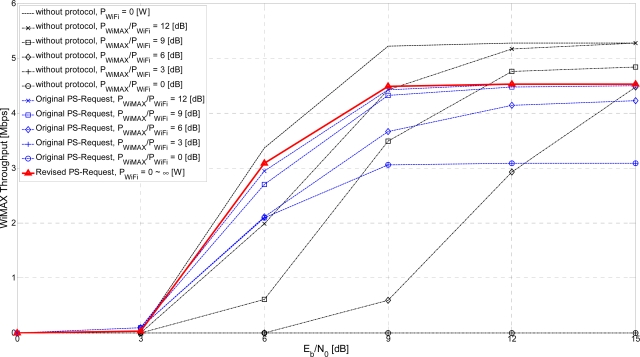
WiMAX throughput *vs*. received *E_b_/N_0_*.

**Figure 12.**
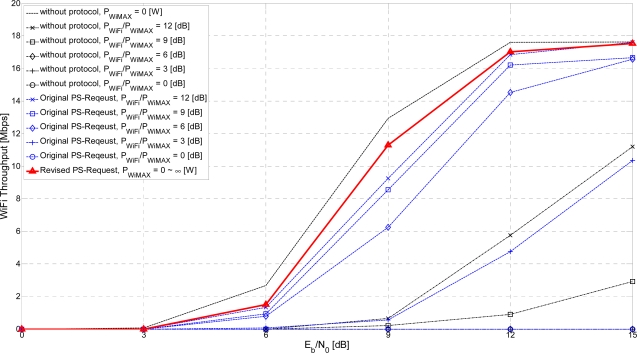
WiFi throughput *vs*. received *E_b_/N_0_*.

Figures 11 and 12 show the simulation results for the following three cases: without the proposed protocols, with the original PS-Request protocol, and with the revised PS-Request protocol. The Signal-to-Interference Ratio (SIR), denoted as *P_interferee_*/*P_interferer_*. *P_interfree_* is the average received signal power of the interferee system, and *P_interferer_* is the average received interference power from the interferer system. The case without the proposed protocols is evaluated for the following six conditions: without interference (*P_interferer_* = 0 [W], which is equivalent to *P_interferee_*/*P_interferer_* = ∞ [dB]), *P_interferee_*/*P_interferer_* = 12 [dB], *P_interferee_*/*P_interferer_* = 9 [dB], *P_interferee_*/*P_interferer_* = 6 [dB], *P_interferee_*/*P_interferer_* = 3 [dB], *P_interferee_*/*P_interferer_* = 0 [dB]. If a WiFi AP can transmit a revised PS-Request frame within a WiFi zone, there will be no mutual interference in the following WiMAX Tx ON period equal to the PSM duration of the WiFi system. That is, *P_interferee_*/*P_interferer_* = ∞ [dB].

Figure 11 shows the throughput of a WiMAX system *vs*. the received *E_b_/N_0_* under interference from a WiFi system sharing the same frequency band. Cases with the proposed PS-Request protocols perform better than the case without the proposed protocols. The performance of the revised PS-Request protocol does not depend on the interference power received from the WiFi system but the received *E_b_/N_0_* of the WiMAX system, differing from the original PS-Request protocol. The revised PS-Request protocol has about 50% more throughput than the original PS-Request protocol for *P_WiMAX_/P_WiFi_* = 0 or 3 [dB] and *E_b_/N_0_* = 12 [dB] at which the throughput for the case without the proposed protocols is zero. Protecting the transmission of WiFi STAs beyond the end of the WiFi zone, as shown in Figure 10, improves performance. For smaller or zero interference environments corresponding to large *P_WiMAX_/P_WiFi_*, the proposed PS-Request protocols may have worse performance, such as smaller throughput, because the allowed transmission interval for the WiFi system cannot be used to transmit data by the WiMAX system. However, for larger interference environments, corresponding to small *P_WiMAX_/P_WiFi_*, the proposed PS-Request protocols can have better performance, such as relatively higher throughput. For the worst case of *P_WiMAX_* = *P_WiFi_* corresponding to *P_WiMAX_/P_WiFi_* = 0 [dB] at which the WiMAX throughput, the WiFi throughput and the sum of two throughputs for the case without the proposed protocols are zero, the revised PS-Request protocol has about 50% more WiMAX throughput than the original PS-Request protocol does.

Figure 12 shows the throughput of a WiFi system *vs*. the received *E_b_/N_0_* under the interference from a WiMAX system sharing the same frequency band. The two proposed PS-Request protocols also perform better than the case without the proposed protocols. The performance of the revised PS-Request protocol outperforms the original PS-Request protocol. This also results from avoiding the overlap of the transmission time of the WiFi system and WiMAX system. The performance of the revised PS-Request protocol depends on the received *E_b_/N_0_* of the WiFi system, rather than on the interference power received from the WiMAX system. For smaller or zero interference environments, corresponding to large *P_WiFi_/P_WiMAX_*, the proposed PS-Request protocols have worse performance, such as smaller throughput, because the allowed transmission interval for the WiMAX system cannot be used to transmit data by the WiFi system. However, for larger interference environments, corresponding to small *P_WiFi_/P_WiMAX_*, the proposed PS-Request protocols can have better performance, such as relatively higher throughput. For the worst case of *P_WiFi_* = *P_WiMAX_* corresponding to *P_WiFiX_/P_WiMAX_* = 0 [dB] at which the WiMAX throughput, the WiFi throughput and the sum of two throughputs for the case without the proposed protocols are zero, the WiFi throughput of the revised PS-Request protocol is similar to the WiFi throughput at the interference-free condition for the case without the proposed protocols, even if that of the original PS-Request protocol is still zero.

## 8. Conclusions

In this paper, we introduced the TDO for the coexistence of WiFi and WiMAX systems sharing a frequency band. We proposed a coexistence zone within WiMAX frame structure, and two PS-Request protocols to achieve the TDO. The two proposed PS-Request protocols are based on the vestigial power management bit within the Frame Control field transmitted by a WiFi AP. We showed using computer simulation that they outperform the case without the proposed protocols. The revised PS-Request protocol can resolve a drawback of the original PS-Request protocol. We evaluated and compared the performance for three cases: without the proposed protocols, with the original PS-Request protocol, and with the revised PS-Request protocol.

For smaller or zero interference environments, the proposed PS-Request protocols have worse performance, such as smaller throughput, because the transmission interval allowed to the other system cannot be used to transmit data by one system. However, for larger interference environments, the revised PS-Request protocols have the best performance of the three cases.

As a topic for further study, we plan to upgrade the simulator used to obtain more realistic and accurate performance measures for more combinations of simulation conditions. We also have a plan to solve the coexistence issue of WiFi and 3GPP LTE (Long Term Evolution) systems.

## Figures and Tables

**Table 1.** Types of system for coexistence.

		**Collaboration**	
		**Not collaborative**	**Collaborative**
**Collocation**	**Not collocated**	Out of scope	Figure 1(a)
	**Collocated**		Figure 1(b)

**Table 2.** WiMAX system parameters.

	**Conventional protocol**	**PS-Request protocols**

$N_{\mathrm{CZ}}^{\mathrm{ofdm}}$	0	16
$N_{\mathrm{DL}}^{\mathrm{ofdm}}$	27(=1 + 2 + 2 × 12)	17(=1 + 2 + 2 × 7)
$N_{\mathrm{UL}}^{\mathrm{ofdm}}$	15(=3 × 5)	9(=3 × 3)
*T_TTG_*	87.2 μs	87.2 μs
*T_RTG_*	74.4 μs	74.4 μs
$N_{\mathrm{WiMAX}}^{\mathrm{slot},1}$	360	180
$N_{\mathrm{WiMAX}}^{\mathrm{tile},\;1}$	1050	840

**Table 3.** Simulation conditions.

	**WiFi System (IEEE802.11g)**	**WiMAX System (IEEE802.16e)**

Bandwidth (MHz)	20	8.75
Sampling frequency (MHz)	20	10
Oversampling	1	2
Sample rate (Msps)	20	20
FFT size	64	1,024
Subcarrier allocation	-	PUSC
Modulation	QPSK	QPSK
Pulse shaping filter	Square root raised cosine filter (Roll-off factor = 1.0)	Square root raised cosine filter (Roll-off factor = 1.0)
Channel coding	Convolutional code (R = 1/2, K = 7)	Convolutional code (R = 1/2, K = 7)
Matched filter	Square root raised cosine filter (Roll-off factor = 1.0)	Square root raised cosine filter (Roll-off factor = 1.0)
Channel	AWGN	AWGN
Allocated time interval, not including TTG and RTG	1.8432 ms	2.9952 ms
Allocated time interval, including TTG and RTG	1.8432 ms	3.1568 ms
SIFS	16 μs	-
DIFS	34 μs	-
Frame time	Variable	5 ms
